# Spectral Inverse Quantum (Spectral-IQ) Method for Modeling Mesoporous Systems: Application on Silica Films by FTIR

**DOI:** 10.3390/ijms131215925

**Published:** 2012-11-28

**Authors:** Ana-Maria Putz, Mihai V. Putz

**Affiliations:** 1 Institute of Chemistry Timisoara of the Romanian Academy, 24 Mihai Viteazul Bld, Timişoara, RO-300223, Romania; E-Mail: putzanamaria@yahoo.com; 2 Laboratory of Computational and Structural Physical Chemistry, Biology-Chemistry Department, West University of Timişoara, Pestalozzi Street No.16, Timişoara, RO-300115, Romania

**Keywords:** particle-wave quantum ratio, sol-gel synthesis, FT-IR spectra, TO_4_ IR absorption band, ionic liquid-like cosurfactants

## Abstract

The present work advances the inverse quantum (IQ) structural criterion for ordering and characterizing the porosity of the mesosystems based on the recently advanced ratio of the particle-to-wave nature of quantum objects within the extended Heisenberg uncertainty relationship through employing the quantum fluctuation, both for free and observed quantum scattering information, as computed upon spectral identification of the wave-numbers specific to the maximum of absorption intensity record, and to left-, right- and full-width at the half maximum (FWHM) of the concerned bands of a given compound. It furnishes the hierarchy for classifying the mesoporous systems from more particle-related (porous, tight or ionic bindings) to more wave behavior (free or covalent bindings). This so-called spectral inverse quantum (Spectral-IQ) particle-to-wave assignment was illustrated on spectral measurement of FT-IR (bonding) bands’ assignment for samples synthesized within different basic environment and different thermal treatment on mesoporous materials obtained by sol-gel technique with *n*-dodecyl trimethyl ammonium bromide (DTAB) and cetyltrimethylammonium bromide (CTAB) and of their combination as cosolvents. The results were analyzed in the light of the so-called residual inverse quantum information, accounting for the free binding potency of analyzed samples at drying temperature, and were checked by cross-validation with thermal decomposition techniques by endo-exo thermo correlations at a higher temperature.

## 1. Introduction

It is already a fact that mesoporous sizes can be controlled by varying the chain length of ionic surfactants or by adding organic molecules, “cosolvents”, which act as spacers inside the micelles [1]. These porous biomaterials with large surface areas and large pore volumes make themselves good candidates for drug delivery systems [2–4]. For a typical drug carrier nano-material to be obtained, silica particles were synthesized by sol-gel methods from tetraethylorthosilicate (TEOS), methoxy-ethanol and deionized water in the presence of ammonia or sodium hydroxide as catalyst at room temperature; hexadecyl trimethy ammonium bromide or the n-dodecyl trimethyl ammonium bromide (DTAB) ionic liquids were used as templates; eventually, results show that after calcinations, porous silica nanoparticles have higher specific surface areas than the only dried ones. However, this important issue addresses to what extent such materials can be classified for their structural potency to free or specific binding with pharmaceuticals in order that their carrier features can be activated. Experimentally, morphologic characterization of the nano-surface constitutes the custom criteria, for instance, with the aid of scanning electron microscopy (SEM) or by atomic force microscopy (AFM) analyses, with the help of nitrogen adsorption/desorption isotherms through applying the Brunaumer-Emmett-Teller (BET), by the Barret–Joyner–Halenda (BJH) methods [5,6] or even by observing the thermal stability disordered *vs.* ordered structures [7].

**Figure 1:**
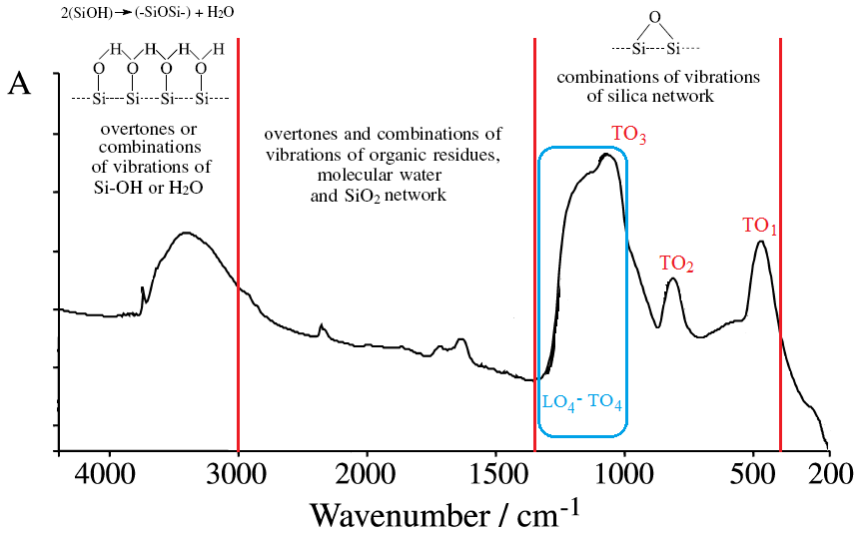
General pattern for wave-number domains of FTIR absorption spectra for silica sol-gel based materials, emphasizing the specific transversal optical (TO) main modes of rocking (TO_1_), symmetric (TO_2_) and antisymmetric (TO_3_) vibrations of oxygen atoms in Si-O-Si bonds along the presently concerned disorder induced longitudinal-transverse vibrational mode (LO_4_-TO_4_) at the frontier of the silica network, along the remaining surface overtones and combinations of the network, residues and water vibrations, respectively. The marked LO_4_-TO_4_ band region is susceptible to wave-particle quantum “phase transition”, thus regulating the physicochemical properties of meso-porosity and bonding at the network surface.

However, when it relates to infrared spectroscopy (IR) investigations on sol-gel silica films [8–10], a part of the consecrated transverse-optical vibrational modes, namely the rocking mode TO_1_ (457–507 cm^−1^) modeling the perpendicular motions of the bridging oxygen to the Si-O-Si plane, the symmetric mode TO_2_ (810–820 cm^−1^) modeling the stretching of oxygen atoms along the bisecting line of the Si-O-Si and the antisymmetric TO_3_ (1070–1250 cm^−1^) describing the motion in opposite distortion of the two neighboring SI-O bonds, there appears to be the so-called disorder-induced TO_4_ modes (about 1200 cm^−1^), interpreted as an increase in bonding strain with a longitudinal-transverse splitting recorded with lower wave numbers of LO (about 1170 cm^−1^) with respect to TO; see Figure 1 and its miss from Table 1[11–25]. However, in this last region within the band 1000–1300 cm^−1^ where the bonding on the surface should be better assigned to the ionic/covalent, porous/free binding or to the particle/wave quantum “phase transition” information, the importance of this assignment resides in the fact that the shown region characterizes the bulk-to-surface physico-chemical richest interaction, beyond which only the overtones and/or combination of vibrations of the network as a whole and of the organic residues and water are dominant; see Figure 1 and Table 1. Therefore, deeper understanding of the TO_4_-LO_4_ “phase transition” region at the frontier of the silica films by FTIR will give crucial information on the porosity of materials at the quantum-to-meso level in view of the hierarchical ordering of materials with higher potential for caring or hosting small molecules in/from organisms or the environment with direct consequences in pharmacology and ecotoxicology [26,27]. Unfortunately, so far, the computational methods available for extracting from experimental spectra such information are missing and in favor of meso-to-macro analysis. Instead, this work makes the advancement of combining the observed data from FTIR spectra with the recent original method of modeling the wave/particle dual information by use of the spectroscopic assignment of the inverse quantum fluctuation factors [28]. This way, the present method fills the quantum-to-meso gap by the so-called *spectral-inverse quantum* (Spectral-IQ) algorithm, lending itself to being generalized and adapted for a wide type of spectra and gas-solid or sol-gel physicochemical interactions.

## 2. Results and Discussion

### 2.1. Spectral-IQ Method

The wave-particle issue was in the “heart” of quantum mechanics, even in its very principles, Heisenberg one in particular; see [28] and references therein. Currently assumed as a complementarity reality, it was just recently quantified with the aim of the path integrals’ quantum fluctuation factor (*n*) through considering the quantum averages for the Gaussian wave packet to the harmonic one for the particle and wave representations, respectively. The results were finite and apart of consistently explaining the atomic (and thus the matter) stability through particle-wave equivalency at the quantum level; they permit also a general formulation of the particle-to-wave ratio content for an observed event [28]:

(1) $${\left(\frac{Particle}{Wave}\right)}_{\begin{array}{l} Observed\\ Evolution \end{array}}=\frac{1}{\sqrt{3+2n^{2}}}\text{exp}\left(\frac{3+n^{2}}{6+4n^{2}}\right)=\{\begin{array}{ll} 0.952 & \ldots n=0\\ 0.667 & \ldots n=1\\ 0 & \ldots n\to \infty \end{array}$$

as well as for the free quantum evolution [28]:

(2) $${\left(\frac{Particle}{Wave}\right)}_{\begin{array}{l} Free\\ Evolution \end{array}}=\frac{1}{\sqrt{3-2n^{2}}}\text{exp}\left(\frac{3-3n^{2}}{6-4n^{2}}\right)=\{\begin{array}{ll} 0.952 & \ldots n=0\\ 1 & \ldots n=0.54909\\ 1.048 & \ldots n=0.87\\ 1 & \ldots n=1\\ \infty & \ldots n=\sqrt{3/2}=1.22474 \end{array}$$

One notes, for instance, that when quantum fluctuations asymptotically increase, the wave contents become infinite and cancel the particle observability, according to the Equation 1.

Instead, even when a system hypothetically experiences zero quantum fluctuations, the wave nature of the system will be still slightly dominant over its particle side at both observed and free evolutions; see the upper branches of Equations 1 and 2. These extremes show that the wave nature of matter will never be fully transferred to particle contents and the mesosystems will never be fully characterized by pure particle (or mechanical) features.

However, it is apparent from Equation 2 that free evolution of a stable system is merely associated with particle dominance, however, without being manifestly observable; in fact, such peculiar particle behavior of the free evolutions of stable matter confirms its inner quantum nature by quantifiable features.

**Figure 2:**
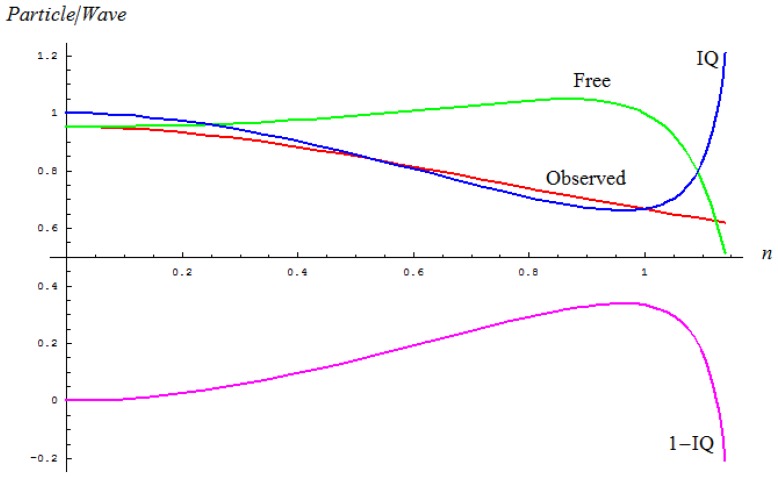
Depicted tendencies of the observed, free and inverse quantum (IQ) evolutions of the particle-to-wave ratio with respect to the quantum fluctuations (*n*) upon Equations 1–3, respectively; the additional curve of residual inverse quantum index *RQ* = 1 − *IQ* was added with the purpose of showing that free evolutions parallels *RQ* that is symmetrical with respect to the IQ factor.

Figure 2 depicts the main tendencies of the particle-to-wave ratios of a stable system in terms of its quantum fluctuation, in free or observed conditions, alongside the present inverse quantum (IQ) index introduced as their competition.

Indeed, the inverse quantum index (3) showcases the manifestly inverse behavior respecting free quantum evolution while accompanying the observed evolution for the respective quantum fluctuations’ range; therefore, it may constitute a suitable index for accounting the particle information degree in a general quantum evolution, from a free-to-observed one. Moreover, if one considers also the residual inverse quantum information *RQ* = 1 − *IQ*, one also gets a symmetrical tool with respect to *IQ* for treating the free evolution at the quantum level.

(3) $${IQ}_{Obs/Free}=\frac{{\left(\frac{Particle}{Wave}\right)}_{\begin{array}{l} Observed\\ Evolution \end{array}}}{{\left(\frac{Particle}{Wave}\right)}_{\begin{array}{l} Free\\ Evolution \end{array}}}=\sqrt{\frac{3-2n^{2}}{3+2n^{2}}}\;\text{exp}\left(\frac{2n^{2}}{9-4n^{4}}\right)$$

Being the quantum fluctuation factor crucial for assessing the free and observed quantum behavior, it should be noted it may discriminate between these two quantum sides of motion, however, based solely on experimental measures of classical and quantum paths, since one considers their squared averages 〈*x*_0_^2^〉*_Exp_* and 〈*x*^2^〉*_Exp_*, respectively, as:

(4) $$n\to n_{Obs}=\sqrt{\frac{{\langle x_{0}^{2}\rangle }_{Exp}}{\left|{\langle x^{2}\rangle }_{Exp}-{\langle x_{0}^{2}\rangle }_{Exp}\right|}}$$

and

(5) $$n\to n_{Free}=\sqrt{\frac{{\langle x_{0}^{2}\rangle }_{Exp}}{{\langle x^{2}\rangle }_{Exp}+{\langle x_{0}^{2}\rangle }_{Exp}}}$$

It is obvious that for a given experimental set-up and records that the resulting observed evolution associates with higher quantum fluctuation than the corresponding free evolution, this feature being consistent with the (extended) Heisenberg uncertainty principle [28].

However, when applied to spectroscopic data, they involve three classes of spectra information in terms of wave-numbers, namely:

- The maximum absorption line wave-number υ̃_0_ (*A*_max_) that relates to the classical path, and the same for squared average measure in the inverse manner as:
  (6) $$\langle x_{0}\rangle =\frac{1}{{\tilde{\upsilon }}_{0}(A_{\text{max}})},$$
  (7) $$\langle x_{0}^{2}\rangle =\frac{1}{{\tilde{\upsilon }}_{0}^{2}(A_{\text{max}})}$$
- The left and right wave-numbers υ̃*_L_*, υ̃*_R_* of the working absorption band, being arithmetically-to-geometrically averaged to get the average of quantum paths “inside” the band:
  (8) $$\langle x\rangle =\frac{\lambda_{L}+\lambda_{R}}{2}=\frac{1}{2}\left(\frac{1}{{\tilde{\upsilon }}_{L}}+\frac{1}{{\tilde{\upsilon }}_{R}}\right)$$
- The full width of a half maximum (FWHM) wave-number Δ υ̃*_FWHM_* of the concerned absorption band that is reciprocally associated with the dispersion of the quantum paths of vibrations within the band:
  (9) $$\Delta x=\frac{1}{\Delta {\tilde{\upsilon }}_{FWHM}}$$

Now, taken together, the quantum averaged path (8) and its dispersion (9) provide the average of the squared quantum paths, according to the general definition [29]:

(10) $$\langle x^{2}\rangle ={\left(\Delta x\right)}^{2}+{\langle x\rangle }^{2}$$

Altogether, the classical and quantum paths’ information of Equations 6–10 inversely correlate to the specific spectroscopic wave-numbers for a given absorption band and correlate the quantum fluctuations’ indices of Equations 4 and 5 with the actual spectral-inverse quantum ones, respectively:

(11) $$n_{Obs}=\frac{1}{{\tilde{\upsilon }}_{0}\sqrt{\frac{1}{\Delta {\tilde{\upsilon }}_{FWHM}^{2}}+\frac{1}{4}\left(\frac{1}{{\tilde{\upsilon }}_{L}^{2}}+\frac{1}{{\tilde{\upsilon }}_{R}^{2}}+\frac{2}{{\tilde{\upsilon }}_{L}{\tilde{\upsilon }}_{R}}\right)-\frac{1}{{\tilde{\upsilon }}_{0}^{2}}}}$$

and

(12) $$n_{Free}=\frac{1}{{\tilde{\upsilon }}_{0}\sqrt{\frac{1}{\Delta {\tilde{\upsilon }}_{FWHM}^{2}}+\frac{1}{4}\left(\frac{1}{{\tilde{\upsilon }}_{L}^{2}}+\frac{1}{{\tilde{\upsilon }}_{R}^{2}}+\frac{2}{{\tilde{\upsilon }}_{L}{\tilde{\upsilon }}_{R}}\right)+\frac{1}{{\tilde{\upsilon }}_{0}^{2}}}}$$

They will be eventually used to compute the observed, free, inverse and residual inverse quantum indices to in depth characterizing of a given material for its porosity-to-free binding ordering through recognizing the particle *vs.* wave quantum tendency of the investigated state by spectroscopy in general and by absorption spectra in the present approach. Specific examples and analyses follow.

### 2.2. Results on Silica Sol-gel-based Mesosystems

**Figure 3:**
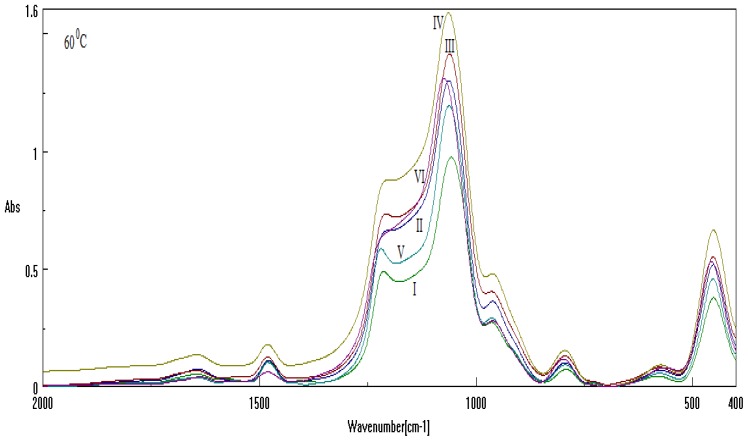
Absorption spectra of samples of Table 2 recorded at 60°C, with the TO_4_ band of Figure 1 enhanced by the respective labeling.

**Figure 4:**
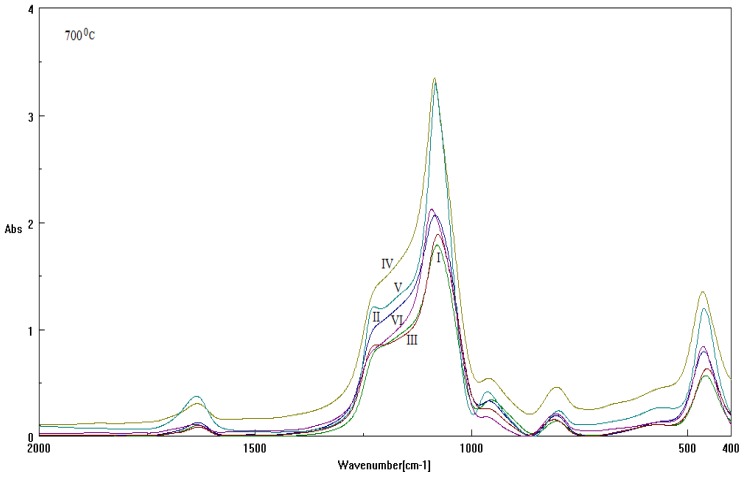
The same spectra records as in Figure 3, here for 700 °C.

Measurement of FT-IR absorption for samples under thermal treatment [30–32], e.g., same ionic liquid chain length, Cetyltrimethylammonium bromide (CTAB), respectively with DTAB or with their combination CTAB+DTAB, in different basic environment, are summarized in the Table 2, and are reported in Figures 3 and 4 for analysis at 60 °C and 700 °C, respectively (refer also to the Experimental Section).

The numerical Spectral-IQ results, as abstracted from Figures 3 and 4, are presented in Tables 3 and 4, for the particle-to-wave (P/W) ratio values in observed and free evolutions, Equations 1 and 2, as based on the quantum fluctuation factors of Equations 11 and 12, along the inverse quantum ratio of Equation 3, respectively. Accordingly, one clearly observes the almost particle-to-wave equivalence throughout all samples, although the residual inverse quantum information 1 – *IQ* makes the significant difference (in some cases, to adouble extent) in the wave- or free-binding content of samples; see for instance I-60 and V-60 with respect to II-60, IV-60 and VI-60 for samples at investigated at 60 °C, and IV-700 *vs.* I-700, V-700 *vs.* III-700 and VI-700 *vs.* II-700 for samples investigated at 700 °C, respectively.

However, in aiming to establish a hierarchy in binding potency, one should run on the residual IQ of the samples for identifying the decreased potency of free bindings information. Accordingly, for 60 °C, one notices from Table 3 the main series VI > IV > II followed by III > V > I, indicating two important features:

- Both series contain all CTAB, DTAB and their combinations;
- The used basic environment is the discriminating factor, here NaOH leaving with more free binding (and less porosity) potential for further interaction than NH_3_, most probably due to the OH group ability (reactivity) to be further involved (and therefore blocked) in the Si surface through the related vibrations and overtones’ combinations; see Figure 1.

Nevertheless, these binding potency series are apparently changing with the rising of the samples’ temperature, as results in Table 4 provide for the 700 °C case; however, agreement with 60 °C is to be researched, while considering specific thermal analysis, as will be exposed later.

### 2.3. Discussion: Cross-Check by Thermal Analysis

**Figure 5:**
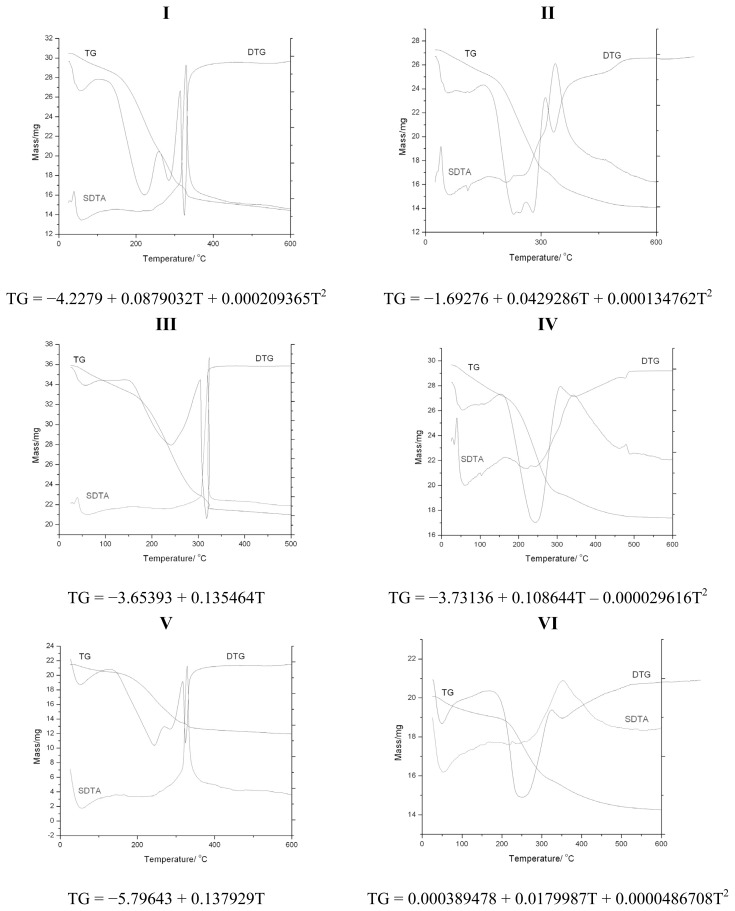
Experimentally obtained thermo-gravimetric (TG), derivative thermo-gravimetric (DTG) and the second derivative thermo-gravimetric analysis (SDTA) curves for the samples of Table 2, along the parabola- and line-like regression curves *TG*[%] = *f* (*T*[^0^*C*]) when based on three and two thermodynamic points of Table 5, including endothermic and exothermic endpoints on the interpolated interval, respectively.

Samples of Table 2 were considered for thermal decomposition treatment, see Figure 5 and the Experimental Section below. The resulting thermo-gravimetric data are summarized in Table 5, as abstracted from Figure 5, while the corresponding correlation curves *TG*[%] = *f*(*T*[^0^*C*]), either as parabola or lines, depending on the number of temperature change points used, three or two, respectively, are provided for each of the samples of Table 2.

These regression curves, while recovering at least the mass loss at the ending temperatures on the interpolation domains, served to provide the missing TG_700_[%] information, *i.e.*, for characterizing the percent of mass loss by rising temperature from 60 to 700 °C; for samples of Table 2, see Table 6. They are to be compared with the information obtained from the recorded spectra of Figure 4 and the allied residual inverse quantum information (1 − *IQ*) of Table 4.

However, to this aim, the relative residual IQ index is computed through containing the relative behavior with respect to the 60 °C data of Table 3; it is thus considered as:

(13) $$R_{1-IQ}=\left|\frac{{\left(1-IQ\right)}_{700}-{\left(1-IQ\right)}_{60}}{{\left(1-IQ\right)}_{60}}\right|$$

with the corresponding values for the samples of Table 2 by the residual IQs from Tables 3 and 4; the results are reported in Table 6. It is worth noting that, since Equation 13 models the amount of relative decrease of the residual of the inverse quantum particle-to-wave information, it naturally correlates with the particle and, thus, to the mass losing amount at 700 °C; thus, it may be compared with the mass loss as provided by thermal treatment.

However, warnings should be made, since the thermal decomposition method belongs merely to classical characterization of the structure with respect to the spectroscopic records of quantum motions (IR vibration in this work); therefore, the comparison is only meaningful unless it is not taken as a one-to-one correspondence. In fact, this is the reason for which, in Table 6, the percent values of Equation (13) were displayed along the TG extrapolated values at 700 °C upon the interpolation curves of Figure 5, here aiming for only a semi-quantitative cross-check.

Nevertheless, if one hierarchically arranges the samples’ increasing absolute differences of Table 6, you will get the series II→VI→IV followed by the succession I→V→III; remarkably, they correspond with the classification provided by (1-IQ) residual analysis of the samples’ spectra at 60°C drying synthesis conditions; see the previous sub-section.

However, both analyses tell us that the most reliable compound for free bindings and, thus, with less potential as a particle carrier, is predicted for the sample VI of Table 2; it is followed by sample IV at drying condition or by sample II under higher temperature circumstances; instead, the silica films obtained within ammonia catalyst (samples I, V and III) are more reliable for being further engaged in the effectors’ interaction due to higher porosity or particle quantum information contained therein; see their higher IQ in Table 3 and 4. Interestingly, the interchange in the last series between samples III and IV for IQ porosity in Table 4 at higher temperature treatment is probably due to the difference in which the shorter chain of DTAB (respecting CTAB) interacts with basic cosolvent at elevated thermal conditions.

All in all, the present spectral-IQ method provides a consistent structural tool in analyzing the spectra for their particle-to-wave quantum content, in the view of establishing the porosity and free binding potential, at various temperatures, respectively, having the silica sol-gel based films as the current working example. Further applications and illustrations of the present method will help in generalizing it towards an in-depth understanding of the quantum influence on the chemical binding potency by physical analysis (spectral, thermal, *etc.*).

## 3. Experimental Section

Porous silica nanoparticles were synthesized by a modified procedure of Tan and co-workers [4] by using HexaDECYL trimethy ammonium bromide and/or n-dodecyl trimethyl ammonium bromide as the porogen and methoxyethanol as the cosolvent. Typically, CTAB or DTAB, or both of them (CTAB plus DTAB), were dissolved in distilled water. Afterwards, ammonia (or NaOH) and methoxyethanol were added; the mixture was vigorously stirred in a closed vessel at room temperature. Then, the sol-gel precursor tetraethyl orthosilicate (TEOS) was dripped into the mixture slowly, by stirring, and the resulting mixture was vigorously stirred for a further 24 h. A white precipitate was collected by centrifugation, which was previously washed with distilled water in an ultrasonic bath, until pH = 7 of the supernatant was reached. Ethanol was added to the precipitate and left for another 24 h, then dried at 40 °C (3h) and to 60 °C (9 h). Template removal (and other organic components) was carried out in air at 700 °C with 2 °C/min rate for 6 h by calcinations (for one half from each sample’s amount).

The thermogravimetric (TG) and differential thermal analyses (DTA) were recorded on a 851-LF 1100-Mettler Toledo apparatus in air flow using alumina crucible. For this purpose, a small sample (mass 29 mg) was placed in the equipment cell and heated from room temperature to 1000 °C at a heating rate of 5 °C min^−1^. Since the disordered structure has higher thermal stability than the ordered one [7], in samples II, IV and VI, the ordering disappears after heating at high temperatures and is more thermally stable compared with the others three samples (I, III, V) of Table 2. The transformations at increasing temperatures of the synthesized samples were captured by thermogravimetry with the recorded curves presented in Figure 5. In the case of *sample I*, the thermo-gram results exhibited three distinct transformations: 60 °C, 260 °C and 360 °C. Thermal gravimetric and differential thermal analyses (TGA and DTA) in air of *sample I* show total weight losses of 47.58%. At 60 °C, TGA registers a weight loss of 1.8% accompanied by an endothermic DTA peak because of the water desorption [33]. At 260 °C, TGA registers a 32.78% weight loss accompanied by an exothermic DTA peak. This is followed by an exothermic transformation at the temperature required, 360 °C, to remove lower-molecular-weight cationic surfactant molecules from the channels [34,35]. Derivative thermogravimetry (DTG) and differential thermal analyses (DTA) of *sample II* show five weight loss steps in the TGA curve, 40 °C, 110 °C, 210 °C, 340 °C and 460 °C, with a total weight loss of 46.57%. At 460 °C, TGA registers a 46.57% weight loss accompanied by an exothermic DTA peak because of the removal of the surfactant. This is consistent with the strong interactions expected between cationic CTAB and silica [35]. In the case of *sample III*, the thermo-gram obtained exhibited two distinct transformations: 30 °C and 310 °C. The endothermic loss near 30 °C (0.41% weight loss) is assigned to water desorption, and at 310 °C (38.34% weight loss), the exothermic transformation takes place because of surfactant removal. In the case of *sample IV*, the thermo-gram displays five distinct transformations: 40 °C, 160 °C, 230 °C, 350 °C and 470 °C. The endothermic loss near 40 °C (0.567% weight loss) is assigned to water desorption, while at 410 °C (40.789% weight loss), the exothermic transformation accompanies the surfactant removal. In the case of *sample V*, the thermo-gram showcases two distinct transformations: 50 °C (1.1% weight loss) and 330 °C (39.72% weight loss), whereas in the case of *sample VI*, the thermo-gram resumes three distinct transformations: 50 °C (1.02% weight loss), 350 °C and 610 °C (29.09% weight loss).

## 4. Conclusions

The present article systemizes the empirical knowledge of correlating mesosystems’ porosity and of their drug carrier potential with the ionic surfactants, co-surfactants and co-solvents, by advancing analytic quantum tools in deciding and ordering such features (here, for silica films-based sol-gel synthesis) in terms of their porosity-to-free binding potential by employing their structural spectra (here, as FTIR); the so-called inverse quantum (IQ) ratio between the observed and free particle-to-wave ratios were accordingly considered in terms of observed and free evolution quantum fluctuation factors, at their turn abstracted from experimentally recorded spectra, upon natural rules involving the wave-number for the maximum line of absorption, along the left-, right- and full-width at half maximum wave-numbers of a concerned band (here, the transversal optical one associated with the induced disorder at the surface of the silica films observed by IR spectroscopy). Comparative analysis based on this IQ factor, as well as on its residual one, 1 − IQ, showcases that, among a CTAB, DTAB and of their combination samples in various basic co-solvents, the simple CTAB+ammonia co-solvent provides the best porosity system for potentially carrying particles and effector interaction in various eco- and bio- nvironments; on the other extreme, the silica film obtained by the cosurfactant combination of CTAB+DTAB in NaOH basicity displays the highest free binding feature, thus being less specific and more associated with environmental hazard to be avoided. This hierarchy was validated by the thermo-gravimetric (TG) analysis, which provided a useful cross-check by interpolating and then extrapolating the TG curves of mass lost against the temperature’s turning points to the desiderate one, to be then compared with the spectral-IQ information. The resulted method is, however, general, based on fundamental particle-to-wave dual quantum behavior; note that the present approach is based on the departure (then associated with the extended Heisenberg uncertainty) between the particle and psi-function description by the ratio between averages of Gaussian to stationary waves, further corresponding to the ratio between the real and the imaginary descriptions of the quantum objects, respectively [28]. Yet, considering the transformation of such a ratio to its nominator-denominator difference, the resulting “physical space” may be associated with the recently introduced inertons—a particle surrounded with its cloud of spatial excitations [36], able to explain the photonic structure and the light-matter interaction in a deeper mechanistic (*i.e.*, deterministic) way; however, such a picture can be completely achieved when the scattered bonding (psi-function) states are also consistently described by their associated quantum particle—the recently introduced bondon [37,38]—so that the inter-particle/bosonic inerton-bondon interaction is finally modeling the obtained/observed spectra. Accordingly, further works are envisaged to validate and to generalize the present spectral-IQ algorithm on various mesosystems [39], while enlarging the cross-check with other available physical experimental methods (SEM, AFM, BET, *etc.*).

## Figures and Tables

**Table 1:** The summary list of the vibration frequencies and their assignments on several bands corresponding to various structural units of the prototype silica network’s FTIR spectra of Figure 1.

cm^−1^	Assignments of the IR vibrations	References
3470–3450	Overlapping of the O-H stretching bands of hydrogen-bonded water molecules (H–O–H···H) with SiO–H stretchings of surface silanols hydrogen-bonded to molecular water (SiO–H···H_2_O)	[11]
3460–3000	Molecular water hydrogens bonded to each other and to SiOH groups; the bands are mainly due to overtones or to combinations of vibrations of Si–OH or H_2_O	[12,13]
3360	The water absorption bands	[13]
3260	Silanol groups absorption bands	[13]
3000–2800	Stretching of the C–H bond within various organic groups	[14,15]
3000–2800	Symmetric and asymmetric fundamental stretching vibrations of CH_2_ and CH_3_ groups belonging to alkoxide and solvent residues	[12]
3000–1350	Overtones and combinations of vibrations of organic residues, molecular water and SiO_2_ network	[12]
2960–2940	Asymmetric stretching of C–CH_3_ and symmetric N–CH_3_ stretching vibration	[16]
2960	Asymmetric C–H stretching of methyl [CH_3_] group of CTAB molecules, if present	[17]
2927	Stretching of the C–H within surfactant (it disappears in the calcinated samples)	[15,18]
2925	Absorption bands due to the CTAB surfactant	[13]
2922–2920	Asymmetric stretching vibration of C–CH_2_ from the methylene chain	[16,17]
2890	Vibration for (CH_3_) can be used to identify the presence of vibrational modes of unreacted TEOS and ethanol in the silica films	[19]
2872	Symmetric stretching vibration of C–CH_3_	[16]
~2850	Absorption bands due to the CTAB surfactant; symmetric C–H stretching of methylene [CH_2_] chain in CTAB	[13,16,17]
2259	A CN stretch	[14,15]
1870–960, 1640–960	Combination of vibrations of the SiO_2_ network (1640 cm^−1^ band is often hidden by molecular water band)	[12]
1640	Molecular water band	[13]
1630–1620	Vibrations of molecular water (present only in the calcinated samples, if it is the case)	[12]
1478	C–H bending of the surfactant (it disappears in the calcinated samples)	[15,18]
~1470	A strong methylene/methyl band (indicative of a long-chain linear aliphatic structure and a high degree of regularity for the linear backbone structure)	[20]
~1350	Absorption band due to the CTAB surfactant	[13]
1350–500	Vibrations of C–H bonds	[12,21]
1300–400	Combinations of vibrations of silica network	[12,21]
1260–1000	Asymmetric stretching vibrations of Si–O–Si bridging sequences	[12,21]
1200–850	Stretching vibration of structural groups containing SiO_4_ tetrahedral	[21–25]
1115–1000	Band for formation of the siloxane bond	[15]
1070	Asymmetric stretching vibrations of Si–O–Si in transverse optical mode (ASTO)	[12]
1050–900, 980–900	Stretching vibration of free silanol groups on the surface of the amorphous solid	[12]
965	Vibration for H_3_CO, can be used to identify the presence of vibrational modes of unreacted TEOS and ethanol in the silica films	[19]
960	Si-OH stretching mode typical of gel structure (decreases in intensity till becoming insignificant when the material undergoes a polycondensation process during drying)	[12]
950	Si–OH bending vibration modes	[13]
929	When identified for CH_2_ can be used to identify the presence of vibrational modes of unreacted TEOS and ethanol in the silica films	[19]
820–800	Symmetric stretching vibrations of Si–O–Si bonds belonging to ring structures	[12]
810	Symmetric Si–O–Si motion	[12]
793	Identified for Si–O and C–O, it can be used to recognize the presence of vibration modes of unreacted TEOS and ethanol in the silica films	[19]
789	Indicates the organic groups as intact through being ascribed to their C–H stretching	[15]
725–720	Methylene rocking vibration (indicative of a long-chain linear aliphatic structure and of a high degree of regularity for the linear backbone structure)	[20]
460–450	Associated with Si–O–Si bond bending (rocking) vibration	[12,21]

**Table 2:** Cases of the ionic liquid-based sol-gel synthesis used in this work. All chemicals were commercially available: Tetraethyl orthosilicate (TEOS), Metoxy-ethanol NH_4_OH (25%), NaOH, CTAB (Cetyltrimethylammonium bromide), and DTAB (*n*-dodecyl trimethyl ammonium bromide).

Sample	Template	Base	Cosolvent	Solution
I	CTAB	NH_3_	Metoxy-ethanol	TEOS
II	CTAB	NaOH		
III	DTAB	NH_3_		
IV	DTAB	NaOH		
V	CTAB+DTAB	NH_3_		
VI	CTAB+DTAB	NaOH		

**Table 3:** The Spectral-IQ results, as based on Equations (1)–(3) with quantum fluctuation factors (11) and (12) for the TO_4_ bands of Figure 3 (υ̃*_L_* = 1299.787[*cm*^−1^], υ̃*_R_* = 999.910[*cm*^−1^]) at 60 °C.

**Sample**	υ̃_0_ (*A*_max_)	Δυ̃*_FWHM_*	*n_Obs_*	(*P*/*W*)*_Evolution_^Observed^*	*n_Free_*	(*P*/*W*)*_Evolution_^Free^*	*IQ*	1 – *IQ*
I-60	1057.76	79.4905	0.0751762	0.94921	0.0747549	0.952777	0.996257	0.00374301
II-60	1063.55	107.4623	0.1011	0.947056	0.100083	0.95348	0.993266	0.00673417
III-60	1061.62	91.0061	0.0857609	0.948407	0.085137	0.95304	0.995138	0.00486168
IV-60	1063.55	112.1339	0.105501	0.946633	0.104346	0.953619	0.992674	0.00732553
V-60	1062.59	80.1571	0.0754605	0.94919	0.0750345	0.952783	0.996229	0.00377119
VI-60	1074.16	117.5866	0.109532	0.946227	0.108241	0.95375	0.992112	0.00788823

**Table 4:** The same type of Spectral-IQ results as in Table 3, here for the TO_4_ bands of Figure 4 (υ̃*_L_* = 1299.787[*cm*^−1^], υ̃*_R_* = 999.910[*cm*^−1^]) at 700 °C.

**Sample**	υ̃_0_ (*A*_max_)	Δυ̃*_FWHM_*	*n_Obs_*	(*P*/*W*)*_Evolution_^Observed^*	*n_Free_*	(*P*/*W*)*_Evolution_^Free^*	*IQ*	1 – *IQ*
I-700	1079.94	126.9705	0.117643	0.945365	0.116048	0.954029	0.990919	0.00908062
II-700	1083.8	156.5567	0.144573	0.942084	0.141643	0.955078	0.986395	0.0136049
III-700	1078.01	94.9767	0.0881346	0.948212	0.0874579	0.953104	0.994868	0.0051321
IV-700	1085.73	87.0955	0.0802383	0.948839	0.0797267	0.952899	0.99574	0.0042602
V-700	1083.8	70.1104	0.0647003	0.949903	0.0644312	0.952549	0.997223	0.00277721
VI-700	1092.48	101.4627	0.0929001	0.947807	0.0921086	0.953237	0.994304	0.00569644

**Table 5:** Identification of the thermo-gravimetric (TG) mass loss (in %) for specific turning points of derivative thermo-gravimetry (DTG) and the associated thermodynamic analysis (DTA), as resulted from the recorded plots of Figure 5 by reading the derivative (D) and second derivative (SD) thermo-gravimetric curves for the samples of Table 2.

Sample	TG mass loss [%]	DTG [°C]	DTA
I	1.80	60	^*^endothermic
	32.78	260	^*^endothermic
	47.58	330	^*^exothermic
II	0.24	40	^*^endothermic
	4.66	110	^*^endothermic
	13.51	210	endothermic
	40.6	340	*exothermic*
	46.57	460	^*^exothermic
III	0.41	30	^*^endothermic
	38.34	310	^*^exothermic
IV	0.567	40	^*^endothermic
	8.49	160	*exothermic*
	19.69	230	^*^endothermic
	36.84	350	*exothermic*
	40.789	470	^*^exothermic
V	1.1	50	^*^endothermic
	39.72	330	^*^exothermic
VI	1.022	50	^*^endothermic
	22.947	350	*exothermic*
	29.09	610	^*^exothermic

“^*^”: marks used points for interpolations on Figure 5.

**Table 6:** The indices of relative residual inverse quantum (IQ) spectral information and the thermo-gravimetrical one as computed upon Equation 13 with the data of Tables 3 and 4, and based on the regression equations of Figure 5, for the samples of Table 2 considered for thermal treatment at 700 °C relative to the drying synthesis case of 60 °C, respectively.

Index	I	II	III	IV	V	VI
R_1-IQ_[%]	142.602	102.028	5.56227	41.8445	26.3572	27.7856
TG_700_[%]	159.893	94.3906	91.1711	57.8074	90.7536	36.4482
